# 20 years on: the legacy of Daksha Emson for perinatal psychiatry

**DOI:** 10.1007/s00737-021-01146-z

**Published:** 2021-06-29

**Authors:** Marisa Casanova Dias, Ekin Sönmez Güngör, Clare Dolman, Livia De Picker, Ian Jones

**Affiliations:** 1 National Centre for Mental Health, MRC Centre for Neuropsychiatric Genetics and Genomics, Cardiff University, Hadyn Ellis Building, Maindy Road, Cardiff, CF24 4HQ UK; 2 Section of Women’s Mental Health, Institute of Psychiatry, Psychology and Neurosciences, King’s College London, London, UK; 3 Erenkoy Mental Health and Neurological Diseases Training and Research Hospital, University of Health Sciences, Istanbul, Turkey; 4 Collaborative Antwerp Psychiatric Research Institute, University of Antwerp, Antwerp, Belgium; 5 University Psychiatric Hospital Campus Duffel, Antwerp, Belgium

**Keywords:** Postpartum psychosis, Bipolar disorder, Suicide, Perinatal mental health services, Perinatal mental health training

## Abstract

The tragedy of Daksha’s death illustrates both the importance of perinatal mental health and the stigma associated with doctors seeking help. With this letter, we express our hope that the lasting legacy of her and others’ tragic stories lies in the continuing improvement and worldwide expansion of perinatal psychiatric services and training so that those in greatest need receive the best care possible wherever — and whoever — they are.

The tragedy of Daksha Emson in October 2000 illustrates both the importance of perinatal mental health and the stigma associated with doctors seeking help (Emson 2004a). Daksha, a Tanzania-born daughter of Indian parents, moved to the UK at the age of nine and entered medical school in 1984 (North East London Strategic Health Authority 2003). During her first year, at the age of 18, she was diagnosed with depression following an extremely serious suicide attempt. Later on, her diagnosis was revised to bipolar disorder, for which she was hospitalized five times and treated with three courses of electro-convulsive therapy (North East London Strategic Health Authority 2003). Despite the impact of these on her studies, she won several prestigious prizes. She married in 1992 with some opposition from family, as noted by the Inquiry into her death, and continued her studies choosing postgraduate training in psychiatry. For the next 8 years and while taking medication (lithium and, at some point, fluoxetine), she never experienced a relapse. Her husband remarks that she was a respected scholar and clinician (Emson 2004b). However, this long period of remission was disrupted when she, after discussing with her psychiatrist, stopped her medication to conceive and breastfeed her baby daughter, Freya, who was born in July 2000. While trying to catch up with her work- and family-related commitments, in October 2000, she suffered an episode of postpartum psychosis. It tragically led her to take the life of her 3-month-old baby daughter, Freya, and then her own, through stabbing and covering both of them in a flammable substance and setting it alight. This happened the day before she was due to resume medication (Emson 2004b). During the previous month, she had been in touch with the community psychiatry nurse and psychiatrist due to poor sleep, and later depression, but she opted to manage without medication in order to continue breastfeeding, as breastfeeding was so important to her (North East London Strategic Health Authority 2003).

The Independent Inquiry to her case emphasized the extended suicide was predisposed by bipolar affective disorder, precipitated by biological factors related to the postpartum period while unmedicated, and also noted the influence of psychosocial stressors (North East London Strategic Health Authority 2003). Isolation, family-related issues, history of miscarriages, stigma, poor perinatal mental health knowledge and organization of services, and other factors may have contributed to this very unfortunate outcome, despite clinical staff acting in good faith. For instance, she was not seen as a formal patient of the community mental health team to protect her anonymity in the NHS. In contrast, sometimes she was seen unofficially, on other occasions few or no notes were taken, and there was no written assessment of her needs nor discussion with the multidisciplinary team. Although she was open about her illness to the occupational health services, she received no help or support from them (Singh 2003). She was systematically failed by the health service and, according to the Inquiry, received “significantly poorer standard of care than that which her own patients might have expected.”

As her husband David predicted, the Independent Inquiry into her care and treatment would “enable Daksha, even in death, to have a positive impact on the care and treatment of other mothers suffering with bipolar illness, and also on the care and treatment of other healthcare workers, stigmatised because of their diagnosed mental illness” (Emson 2004a).

Twenty years after the death of a 34-year-old bright psychiatrist, her story and similar tragic cases have had a profound impact:

(i) On the development of specialist perinatal mental health services (Howard and Khalifeh 2020);
(ii) On initiatives such as perinatal psychotropic information services (http://www.ppmis.org.au/) and peer support (https://www.app-network.org/peer-support/);
(iii) On the founding of third sector organizations such as Action on Postpartum Psychosis and the Maternal Mental Health Alliance in the UK;
(iv) On the establishment of international umbrella organizations such as the Global Alliance for Maternal Mental Health (https://globalalliancematernalmentalhealth.org/);
(v) On the recognition of risk in women with bipolar disorder and their need for preconception advice (Dolman et al. 2016; Jones and Craddock 2005);
(vi) On the development of specialized confidential services for health professionals, removing some of the stigma attached to help-seeking (Gerada 2019).

The impact of Daksha’s legacy on perinatal psychiatry training should also not be understated. Daksha’s story has been used internationally to increase awareness of the importance of adequate perinatal mental health training. The tragedy of her and her baby’s avoidable deaths is a powerful means of promoting psychiatry to medical students and trainees and as a reminder to psychiatrists of just how critical their interventions — or lack of them — can be.

The Inquiry report stated that, had she lived in a different location, she would have had access to perinatal psychiatric expertise and therefore might still be alive (North East London Strategic Health Authority 2003). There have been changes over the last 20 years with more recent widespread development of perinatal services (see Fig. 1). Specialist community perinatal mental health teams and inpatient services called Mother and Baby Units (National Institute of Health and Care Excellence n.d.) are now provided or promised in all four nations of the UK. In those services, perinatal psychiatrists work in multidisciplinary teams, which usually include psychologists, mental health nurses, social workers, occupational therapists, and nursery nurses, and also work closely with health visitors, midwifes, obstetricians, and general practitioners. Figure 2 illustrates the perinatal mental health care pathways which outline how women can access services. Preconception advice is usually provided by perinatal psychiatrists. Early identification of perinatal mental disorders is done through the universal services of primary care, midwifery/obstetrics, and home-visiting nurses/health visitors. Only the moderate to severe presentations are seen in mental health services, although there is currently a discussion to expand specialist services to less severe and more complex presentations, to include partner assessment, and to extend from 1 to up to 2 years postpartum (Howard and Khalifeh 2020).

**Fig. 1:**
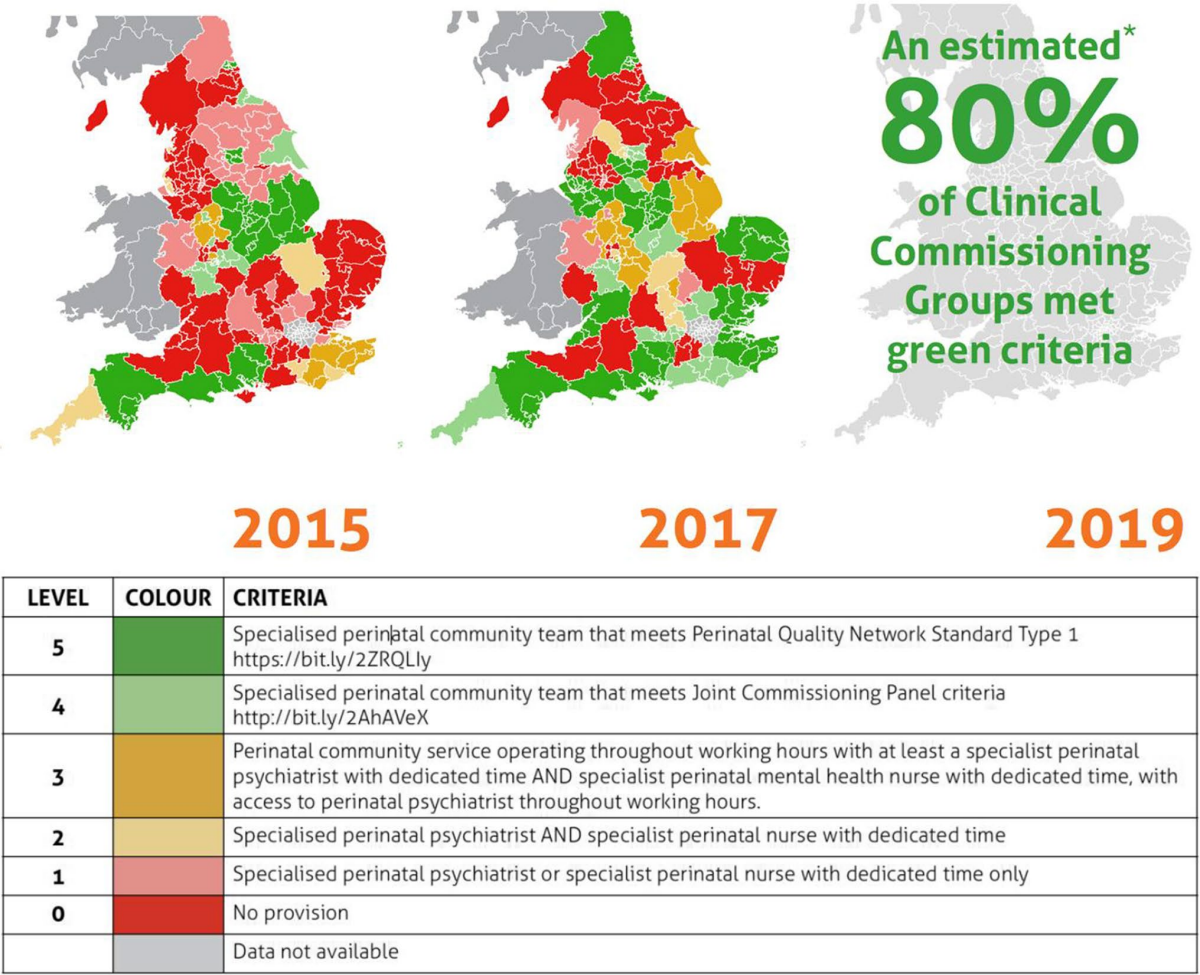
Map of specialist community perinatal mental health teams (England) in the last 5 years. Figure published by the Maternal Mental Health Alliance, 2020. www.maternalmentalhealthalliance.org. *This estimate is based on the data received. The collection and verification of data for England was not complete before COVID-19

**Fig. 2:**
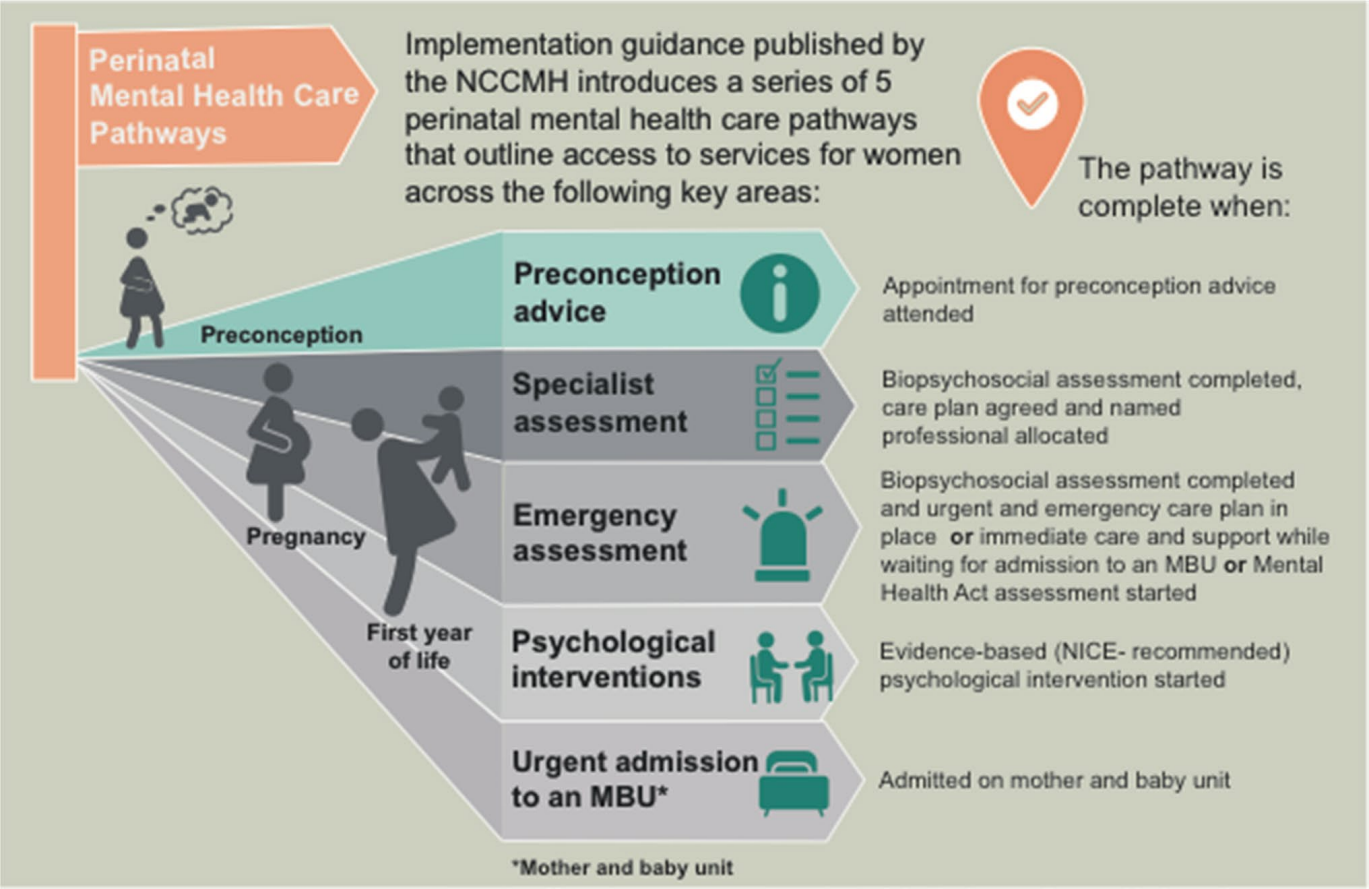
Perinatal mental health care pathways in the UK. Figure adapted from https://www.rcpsych.ac.uk/improving-care/nccmh/care-pathways/perinatal-pathways. NCCMH, National Collaborating Centre for Mental Health; NICE, National Institute for Health and Care Excellence

As a way of promoting the highest level of care, a quality network for perinatal services was established in 2007 to provide accreditation and peer appraisal of services based on a set of standards (Royal College of Psychiatrists n.d.). These standards, which are informed by evidence and best practice, have evolved through time, now including for instance specific reference to peer support workers and involvement of patients and their families. Although this expansion of services in the UK has brought many improvements, little is known currently about which service models work best and a lot of work remains to be done, both within the UK and internationally (Howard and Khalifeh 2020).

With this letter, we express our hope that the lasting legacy of her tragic story lies in the continuing improvement and worldwide expansion of perinatal psychiatric services and training so that those in greatest need receive the best care possible wherever — and whoever — they are.

## Data Availability

Not applicable.
